# Impact of physical activity level and dietary fat content on passive overconsumption of energy in non-obese adults

**DOI:** 10.1186/s12966-017-0473-3

**Published:** 2017-02-06

**Authors:** Kristine Beaulieu, Mark Hopkins, John Blundell, Graham Finlayson

**Affiliations:** 1 School of Psychology, University of Leeds, Leeds, LS2 9JT UK; 2 School of Food Science & Nutrition, University of Leeds, Leeds, LS2 9JT UK

**Keywords:** Habitual physical activity, Appetite control, Passive overconsumption, High-fat, Body composition

## Abstract

**Background:**

Passive overconsumption is the increase in energy intake driven by the high-fat energy-dense food environment. This can be explained in part because dietary fat has a weaker effect on satiation (i.e. process that terminates feeding). Habitually active individuals show improved satiety (i.e. process involved in post-meal suppression of hunger) but any improvement in satiation is unknown. Here we examined whether habitual physical activity mitigates passive overconsumption through enhanced satiation in response to a high-fat meal.

**Methods:**

Twenty-one non-obese individuals with high levels of physical activity (HiPA) and 19 individuals with low levels of physical activity (LoPA) matched for body mass index (mean = 22.8 kg/m^2^) were recruited. Passive overconsumption was assessed by comparing ad libitum energy intake from covertly manipulated high-fat (HFAT; 50% fat) or high-carbohydrate (HCHO; 70% carbohydrate) meals in a randomized crossover design. Habitual physical activity was assessed using SenseWear accelerometers (SWA). Body composition, resting metabolic rate, eating behaviour traits, fasting appetite-related peptides and hedonic food reward were also measured.

**Results:**

In the whole sample, passive overconsumption was observed with greater energy intake at HFAT compared to HCHO (*p* < 0.01), without any differences between activity groups (*p* > 0.05). SWA confirmed that HiPA were more active than LoPA (*p* < 0.01). HiPA had lower body fat and greater fat-free mass than LoPA (*p* < 0.05 for both) but did not differ in resting metabolic rate, eating behaviour traits, appetite-related peptides or food reward (*p* > 0.05 for all).

**Conclusions:**

Non-obese individuals with high or low physical activity levels but matched for BMI showed similar susceptibility to passive overconsumption when consuming an ad libitum high-fat compared to a high-carbohydrate meal. This occurred despite increased total daily energy expenditure and improved body composition in HiPA. Greater differences in body composition and/or physical activity levels may be required to impact on satiation.

**Electronic supplementary material:**

The online version of this article (doi:10.1186/s12966-017-0473-3) contains supplementary material, which is available to authorized users.

## Background

There is abundant evidence to support the benefits of habitual physical activity in weight management [1]. Myers et al. have recently shown significant negative associations between objectively-measured moderate-to-vigorous physical activity and markers of adiposity [2]. On the other side of the energy balance equation, the contribution of high-fat energy-dense foods towards obesity cannot be ignored [3, 4]. Passive overconsumption is a global phenomenon and originates from changes in the food supply towards increasingly energy-dense foods, contributing greatly to the obesity epidemic [5]. This is reflected by an unintentional increase in energy intake, arising from a failure to appropriately adjust intake in response to energy density [6]. Control over food intake is strongly influenced by ingestive and post-ingestive feedback from satiation and satiety, two separate aspects of appetite that inhibit eating [7]. Satiation is the process that terminates feeding, measured by the amount of food eaten at a meal, and satiety is the process involved in post-meal suppression of hunger, often measured with a preload-test meal paradigm using preloads differing in energy content [7]. The satiety quotient (SQ), calculated from changes in appetite scores relative to a meal’s energy content [8], can also provide a measure of satiation (immediately after food consumption) and satiety (over a specified amount of time after food consumption) [9]. Dietary fat exerts a weaker effect on satiation within a meal than carbohydrate or protein, and is a key driver of passive overconsumption [6]. For example, in the short-term, when eating ad libitum and to a comfortable level of fullness, individuals consume more calories from high-fat foods compared to high-carbohydrate foods [10–12]. Passive overconsumption is strongly influenced by the higher energy density of fat relative to carbohydrate and protein (9 vs. 4 kcal/g, respectively) [13]. Consequently, eating a high-fat energy-dense diet is conducive to overconsumption and a positive energy balance.

It has been proposed that habitual physical activity improves the sensitivity of the appetite control system [14, 15]. Compared to their inactive counterparts, active individuals decrease their energy intake at an ad libitum test meal following a high-energy preload compared to a low-energy preload [16–19]. However, preload studies preclude us from differentiating between satiation and satiety as separate components of appetite. Additionally, little is known regarding the differences in hedonic mechanisms of appetite control (i.e. food reward and preference for high-fat foods) across different physical activity levels, although research on this topic is emerging [20]. Therefore, this study assessed the satiation response to meals varying in fat and carbohydrate in individuals with high levels of physical activity (HiPA) compared to those with low levels of physical activity (LoPA). It was hypothesized that compared to LoPA, HiPA would: consume less energy in the HFAT condition relative to HCHO condition, show a greater satiation response (SQ), have a reduced hedonic response to high-fat foods in response to HFAT and show lower susceptibility to overconsumption on psychological trait measures.

The study was conducted within a multi-level experimental platform assessing several dimensions of appetite control (e.g. environmental, behavioural, psychological, physiological, and metabolic) [21]. Thus, in addition to measuring the response to passive overconsumption, we sought to examine the effects of physical activity level in several putative determinants of appetite control such as body composition, resting metabolic rate, daily energy expenditure, appetite-related peptides, and eating behaviour traits as secondary outcome measures.

## Methods

### Participants

Forty non-obese adults (21 HiPA and 19 LoPA) aged 18–55 years were recruited via poster and email lists at the University of Leeds, UK (see Table 1 for participant characteristics). Groups were matched for age, sex and body mass index (BMI). Participants were screened for inclusion based on the following criteria: BMI between 20.0 and 29.9 kg/m^2^ (to allow for a range of body composition), non-smoker, weight stable (±2 kg for previous 3 months), no change in physical activity over the previous 6 months, not currently dieting, no history of eating disorders, not taking any medication known to affect metabolism or appetite, and acceptance of the study foods. In addition, the short-form of the validated International Physical Activity Questionnaire [22] was used to screen for physical activity levels, with participants only eligible if they engaged in at least 40 min of moderate-to-vigorous physical activity during 4 days or more per week (HiPA), or less than 40 min of moderate-to-vigorous physical activity during 1 day or less per week (LoPA). These criteria were based on a previous study that demonstrated differences in satiety between exercisers and non-exercisers [16], and have been used in subsequent studies [20, 23]. Habitual physical activity was subsequently measured objectively using tri-axial accelerometry (SenseWear Armband (SWA); BodyMedia, Inc; Pittsburgh, USA). The study was approved by the School of Psychology Ethical Committee at the University of Leeds (15–0181). Participants provided written informed consent prior to taking part and were remunerated on completing the study.

**Table 1:** Group characteristics of HiPA and LoPA participants

	HiPA	LoPA	*p*-value
*n*	20 (10 F)	19 (11 F)	
Age (years)	29.9 ± 9.6	30.4 ± 9.3	0.851
BMI (kg/m^2^)	22.6 ± 1.9	23.1 ± 2.7	0.490
Total mass (kg)	68.2 ± 11.1	64.0 ± 11.9	0.264
Fat mass (kg)	13.1 ± 5.4	16.8 ± 6.0	0.056
Fat-free mass (kg)	55.0 ± 11.9	47.3 ± 8.6	**0.025**
Body fat (%)	19.7 ± 8.2	25.6 ± 7.1	**0.018**
RMR (kcal/day)	1669.8 ± 226.7	1570.9 ± 296.8	0.248
RER	0.79 ± 0.07	0.75 ± 0.06	0.061
WC (cm)	79.8 ± 5.5	81.2 ± 9.4	0.593
VO_2max_ (mL/kg/min)	50.5 ± 7.5	34.7 ± 5.6	<**0.001**
Fasting glucose (mmol/L)	4.8 ± 0.4	5.0 ± 0.4^a^	0.221
Fasting insulin (mU/L)	7.1 ± 3.3	8.7 ± 4.5^a^	0.225
HOMA-IR	1.52 ± 0.74	2.00 ± 1.25^a^	0.166
Fasting leptin (pg/mL)	8033.4 ± 7712.2	8561.2 ± 5743.6^a^	0.821
Fasting ghrelin (pg/mL)	47.2 ± 26.4^b^	71.8 ± 58.9^c^	0.246

^a^ *n* = 16

^b^ *n* = 12

^c^ *n* = 10

*BMI* body mass index, *HOMA*-*IR* homeostasis model of risk assessment-insulin resistance, *HiPA* high level of physical activity, *LoPA* low level of physical activity, *RER* respiratory exchange ratio, *RMR* resting metabolic rate, *VO*_2max_ maximal aerobic capacity, *WC* waist circumference

### Study design

**Fig. 1:**
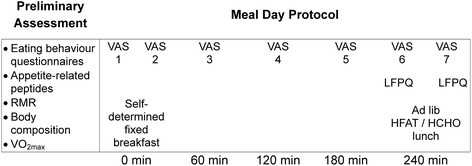
Experimental protocol. HCHO, high-carbohydrate; HFAT, high-fat; LFPQ, Leeds Food Preference Questionnaire; RMR, resting metabolic rate; VAS, visual analogue scales; VO_2max_, maximal aerobic capacity

As seen in Fig. 1, following a preliminary assessment, HiPA and LoPA participants underwent 2 laboratory probe days in a quasi-experimental study that included a self-determined fixed breakfast followed by an ad libitum high-fat (HFAT) or high-carbohydrate (HCHO) lunch meal in a randomized crossover design. For the 48 h prior to the three testing sessions, the participants refrained from exercise, and for the 24 h prior, did not consume caffeine or alcohol. On each test day, the participants arrived at the research unit between 07:00 and 09:00 following a 10-h fast (no food or drink except water). Prior to the first meal day, the participants consumed their habitual diet but were required to record their food intake for 24 h in a diary that was provided to them during the preliminary assessment, and replicated their food intake prior to the subsequent meal day. Compliance with these guidelines were verified upon arrival at the laboratory for each testing session.

During the 2 meal days, measurements included subjective appetite ratings, hedonic preference (explicit liking and implicit wanting) for high-fat foods, and energy intake at breakfast and at an ad libitum HFAT or HCHO lunch 4 h later. Breakfast was ad libitum on the first meal day and then fixed at the same level of intake on the second meal day. At the end of the first meal day, the participants were fitted with the SWA, which was worn for 7 days. Each meal day was separated by at least 9 days.

### Preliminary assessment

Approximately 1 week before the meal days, anthropometrics, body composition (fat mass and fat-free mass), resting metabolic rate, maximal aerobic capacity, eating behaviour traits and fasting appetite-related peptides (leptin, acylated ghrelin, insulin, and glucose) were measured.

### Anthropometrics and body composition

Standing height without shoes was measured using a stadiometer (Leicester height measure, SECA; UK). Fat mass, fat-free mass and percentage body fat were estimated via air displacement plethysmography (BodPod, Life Measurement, Inc.; USA) following the manufacturer’s instructions and using the Siri equation [24]. Body mass was obtained from the BodPod. Waist circumference was measured using a measuring tape at the level of the umbilicus.

### Resting metabolic rate

Resting metabolic rate (RMR) was measured with an indirect calorimeter fitted with a ventilated hood (GEM, Nutren Technology Ltd; UK) following the guidelines of The American Dietetic Association [25]. Participants were required to remain awake but motionless in a supine position for 40 min. The average of the last 30 min of collection was used to determine RMR. Substrate oxidation (respiratory exchange ratio; RER) was calculated using standard stoichiometric equations [26].

### Maximal aerobic capacity

Maximal aerobic capacity (VO_2max_) was determined using a maximal incremental treadmill test based on the modified Balke protocol [27], with the incline increasing 2% in the first minute and 1% for each additional minute until volitional exhaustion. Expired gases (Vyntus CPX, CareFusion; UK) and heart rate (Polar RS400, Polar; Finland) were measured continuously during the test. Prior to each test, the gas analyser was calibrated using gases of known concentrations while the volume sensor was calibrated automatically by the system at flow values of 2 L/s and 0.2 L/s. The average of the last 20 s of the test was considered VO_2max_.

### Eating behaviour traits

Participants completed four validated psychometric questionnaires to assess eating behaviour traits. The Three-Factor Eating Questionnaire (TFEQ) measures three dimensions of eating behaviour: cognitive control of restraint, disinhibition of eating, and susceptibility to hunger [28]. The Binge Eating Scale (BES) assesses the severity of binge eating [29]. The Yale Food Addiction Scale (YFAS) assesses addictive eating behaviour and significant clinical impairment of distress as a result of overeating [30]. The Control of Eating Questionnaire (CoEQ) is designed to assess the severity and type of food cravings experienced over the previous 7 days [31].

### Appetite-related peptides

A fasting blood sample was taken by venepuncture for the assessment of leptin, acylated ghrelin, insulin, and glucose. Blood was drawn in EDTA, serum and fluoride collection tubes. Aprotinin (50 μL/mL blood) was immediately added to the EDTA tube for preservation of ghrelin. Plasma and serum obtained were aliquoted and stored at −70 °C until analysis by the Department of Pathology Research & Development at the Leeds Teaching Hospitals NHS Trust (Leeds, UK). All samples analyses were conducted in one batch. Plasma glucose was analysed with the ADVIA Chemistry Glucose Oxidase Concentrated assay (Siemens Healthcare Diagnostics Inc.; UK), serum insulin with the ADVIA Centaur Insulin assay (Siemens Healthcare Diagnostics Inc.; UK), plasma leptin with the Quantikine Human Leptin Immunoassay ELISA kit (R&D Systems Europe Ltd.; UK) and acylated ghrelin with the Spi Bio Acylated Ghrelin Express Enzyme Immunoassay kit (Bertin Pharma; France). The range for the coefficients of variation for intra-assay precision for glucose, insulin, leptin, and acylated ghrelin are 0.2–0.3%, 3.2–4.6%, 3.0–3.3%, and 5.5–10.3%, respectively. Insulin resistance was calculated via the homeostasis model of risk assessment (HOMA-IR) [32].

### Meal days

#### Test meals

Breakfast during the first meal day was ad libitum with wholegrain cereal, semi-skimmed milk and water served in excess of expected consumption. Coffee or tea was also offered (175 g). Food items were weighed before and after consumption to the nearest 0.1 g and the quantities consumed by each participant were subsequently replicated for the next meal day to make the energy content of the meal individually fixed. Energy intake was calculated using energy equivalents for protein, fat and carbohydrate of 4, 9 and 3.75 kcal/g, respectively, from the manufacturers’ food labels. The participants were allowed to leave the laboratory in between breakfast and lunch but were not allowed to eat or drink any foods except water from the bottle provided.

Lunch was presented in excess of expected consumption and included HFAT or HCHO rice and yoghurt. Water (350 g) was also offered. The ingredients of the meals were covertly manipulated to make them HFAT (1.99 kcal/g, 41.3% carbohydrate, 50.7% fat, 8.0% protein) or HCHO (1.41 kcal/g, 70.8% carbohydrate, 19.5% fat, 9.7% protein) but of similar palatability achieved through pilot testing and confirmed by the participants after each meal (sweetness, savouriness, tastiness, pleasantness; *p* > 0.05 for all). The HFAT rice dish contained tomato & basil rice, vegetable oil, double cream, and grated cheese, and the yoghurt contained low-fat plain yoghurt, double cream, sugar, and maltodextrin. The HCHO rice dish contained tomato & basil rice, vegetable stock, and semi-skimmed milk, and the yoghurt contained whole-milk plain yoghurt, sugar, and maltodextrin. Participants were instructed to eat as little or as much as they wanted until comfortably full. Food items were weighed before and after consumption, and energy intake was calculated as described above. To examine passive overconsumption while accounting for physical activity level, the difference in energy intake between HFAT and HCHO was calculated as a percentage of total daily energy expenditure (TDEE) obtained from the SWA (see methods below), and labelled passive overconsumption index.

### Appetite sensations and hedonic preference for high-fat foods

Subjective appetite sensations were assessed via visual analogue scales for hunger and fullness before and after each meal and at hourly intervals throughout the meal day [33]. The satiety quotient (SQ) [8] was calculated for each condition using energy intake at the respective meals with the following formula:

$$ \mathrm{S}\mathrm{Q}\ \left(\mathrm{mm}/\mathrm{kcal}\right) = \frac{\left(\mathrm{rating}\ \mathrm{before}\ \mathrm{eating}\ \mathrm{episode}\hbox{-} \mathrm{rating}\ \mathrm{after}\ \mathrm{eating}\ \mathrm{episode}\right)}{\mathrm{energy}\ \mathrm{of}\ \mathrm{the}\ \mathrm{food}\ \mathrm{consumed}} \times 100 $$

The Leeds Food Preference Questionnaire was administered before and after lunch to determine scores of implicit wanting and explicit liking for high-fat (>50% energy) and low-fat (<20% energy) foods matched for familiarity, sweetness, protein, and acceptability [34]. Low-fat scores were subtracted from high-fat scores to obtain the fat appeal bias score; thus a positive score indicates greater liking or wanting towards high-fat compared to low-fat foods.

### Free-living physical activity and energy expenditure

Free-living physical activity and energy expenditure were measured using the SWA in between the 2 meal days, as previously described [2]. Briefly, the participants were instructed to wear the armband on their non-dominant arm over 7 days for at least 23 h per day (awake and asleep, except for the time around showering, bathing or swimming). Compliance was defined as 5 days of wear (including 1 weekend day) with at least 22 h of verifiable time per day. Proprietary algorithms available in the accompanying software (version 8.0 professional) were used to calculate TDEE, PAL (physical activity level; TDEE/basal metabolic rate), minutes spent sleeping, sedentary (<1.5 METs) or in light (1.5–2.9 METs), moderate (3.0–5.9 METs) and vigorous (≥6.0 METs) physical activity. The SWA has shown good accuracy in estimating free-living TDEE and various intensities of physical activity [35–37].

### Statistical analysis

Data are reported as mean ± standard deviation. IBM SPSS for Windows (version 21; USA) was used for statistical analyses. We based our sample size after the study by Long et al. [16] who demonstrated in non-obese individuals a difference in food intake between frequent exercisers and non-exercisers. In this preload-test meal design the difference in food intake between groups was ~400 kcal with an effect size of d = 0.94. We allowed for a similar effect size in the present study and calculated that n = 21 per group would be sufficient to detect a difference in intake under the high-fat condition with 1-β = 0.9 and α = 0.05, one-tailed. A total of 39 participants were included in the final sample (HiPA: 10 males, 10 females; LoPA: 8 males, 11 females), as one male participant in HiPA was excluded due to feeling very unwell during the second meal day. Blood samples for 36 participants (20 HiPA and 16 LoPA) were successfully obtained for glucose, insulin and leptin, and because of technical difficulties with the assay, for 22 participants (12 HiPA and 10 LoPA) for ghrelin. SWA data were compliant in 36 participants (19 HiPA and 17 LoPA; 92% compliance). Independent sample *t*-tests were used to determine differences in participant characteristics between LoPA and HiPA groups. Differences in passive overconsumption index between LoPA and HiPA groups were examined with an independent sample *t*-test. Differences in energy intake and SQ were identified with two-way mixed-model ANOVAs, with the between-subject factor of physical activity level (HiPA vs. LoPA) and the within-subject factor of meal condition (HFAT vs. HCHO). Differences in appetite sensations and fat appeal bias were identified with three-way mixed-model ANOVAs, with the between-subject factor of physical activity level (HiPA vs. LoPA) and the within-subject factors of meal condition (HFAT vs. HCHO) and food consumption (pre- vs. post-lunch). Statistical significance was established at *p* < 0.05 and trends were considered at *p* ≤ 0.07.

## Results

### Participant characteristics

Despite there being no group differences in BMI, HiPA had significantly lower body fat and greater fat-free mass and VO_2max_ than LoPA (Table 1). HiPA also had a tendency for lower fat mass and greater RER than LoPA (Table 1). There were no significant differences in eating behaviour traits from the CoEQ, BES, TFEQ or YFAS between HiPA and LoPA (*p* > 0.05 for all; data not shown), but there was a trend towards greater restraint in HiPA (8.8 ± 5.6) compared to LoPA (6.0 ± 3.6; *p* = 0.07).

There were no group differences in minutes of SWA wear time or sleep time (Table 2). HiPA and LoPA differed on objectively measured habitual physical activity (Table 2); HiPA had significantly greater number of daily steps, TDEE, light physical activity, moderate-to-vigorous physical activity, PAL, and lower sedentary behaviour than LoPA.

**Table 2:** Habitual physical activity from the SenseWear Armband

	HiPA^a^	LoPA^b^	*p*-value
SWA wear time (min/day)	1411.9 ± 17.6	1419.2 ± 8.6	0.121
Sleep (min/day)	415.1 ± 26.6	432.1 ± 56.7	0.268
Daily steps	11146.9 ± 4258.9	8236.0 ± 2670.1	**0.019**
TDEE (kcal/day)	2967.8 ± 549.0	2368.3 ± 449.8	**0.001**
Sedentary behaviour (min/day)	515.0 ± 126.4	642.5 ± 100.6	**0.002**
Light PA (min/day)	300.5 ± 83.7	243.0 ± 91.0	0.056
MVPA (min/day)	182.2 ± 67.1	102.8 ± 37.4	<**0.001**
PAL	1.88 ± 0.24	1.55 ± 0.13	<**0.001**

^a^ *n* = 19

^b^ *n* = 17

*HiPA* high level of physical activity, *LoPA* low level of physical activity, *MVPA* moderate-to-vigorous physical activity, *PA* physical activity, *PAL* physical activity level, *SWA* SenseWear Armband, *TDEE* total daily energy expenditure

### Energy intake and passive overconsumption

**Fig. 2:**
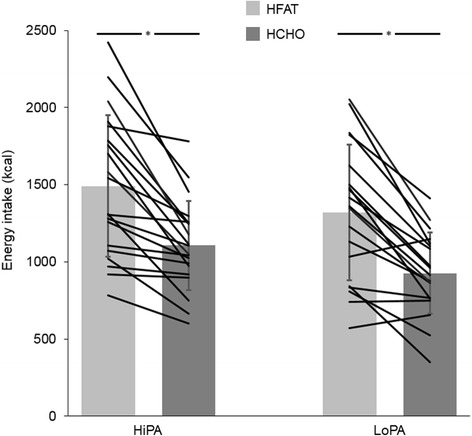
Energy intake at ad libitum high-fat (HFAT) and high-carbohydrate (HCHO) lunch meals. Mean ± standard deviation; *black* lines represent individual responses; *p < 0.001 (main effect of condition HFAT vs. HCHO). HiPA, high level of physical activity; LoPA, low level of physical activity

There were no significant differences in breakfast energy intake between groups (HiPA: 465 ± 208 kcal vs. LoPA: 395 ± 147 kcal; *p* = 0.231) or between meal days (*p* = 0.791). For energy intake at the HFAT and HCHO ad libitum lunch meals (Fig. 2), there was a significant main effect of condition (*p* < 0.001), such that energy intake was higher in HFAT than HCHO, but no main effect of group or condition by group interaction (*p* > 0.05 for both). Full sample results can be found in Additional file 1. There were no significant group differences in the passive overconsumption index (HiPA: 12.8 ± 9.9% vs. LoPA: 16.3 ± 10.8%; *p* = 0.301).

### Appetite ratings and hedonic preference for high-fat foods

**Fig. 3:**
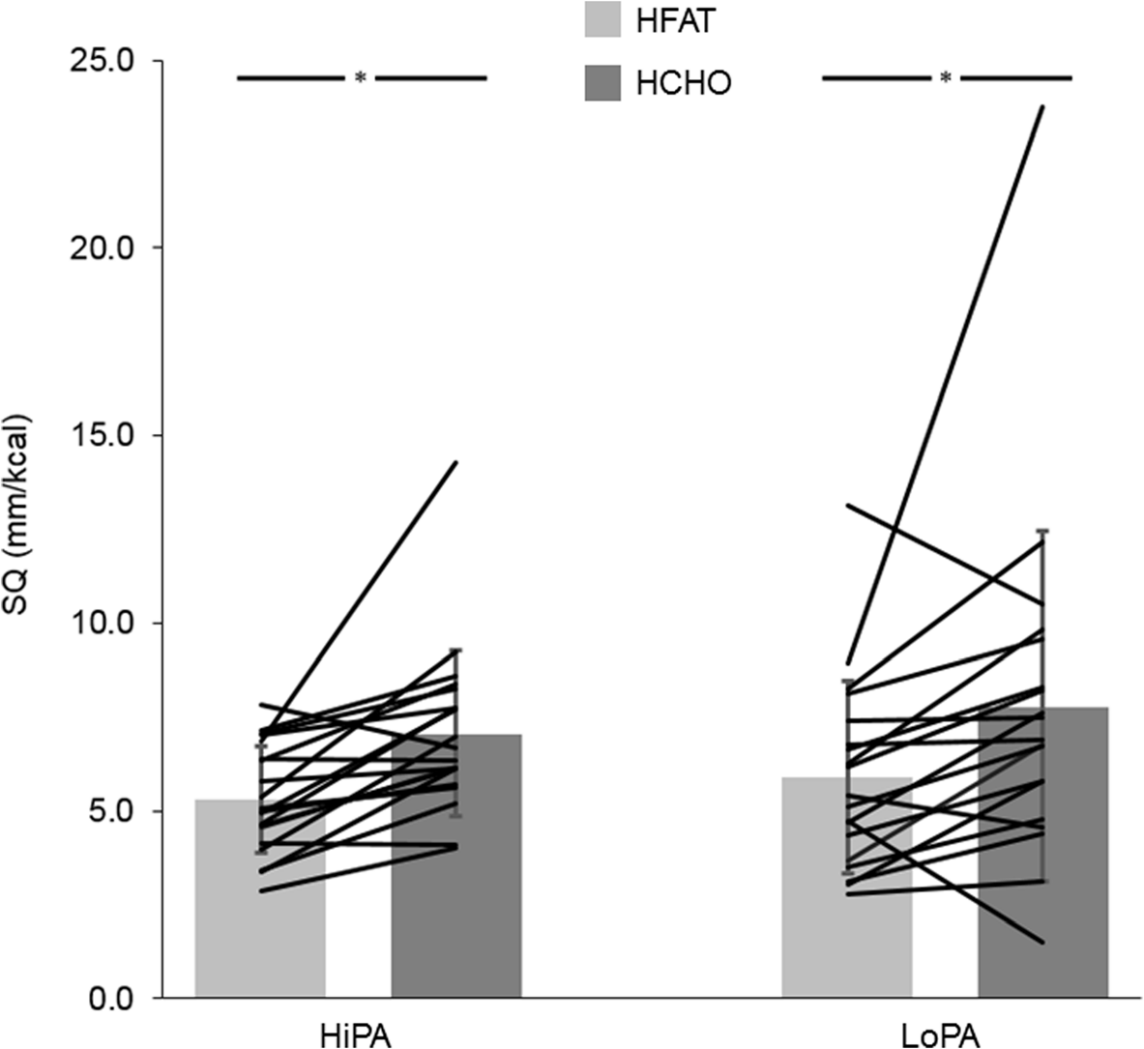
Satiety quotient (SQ) at the ad libitum high-fat (HFAT) and high-carbohydrate (HCHO) lunch meals. Mean ± standard deviation; *black* lines represent individual responses; *p < 0.001 (main effect of condition HFAT vs. HCHO). HiPA, high level of physical activity; LoPA, low level of physical activity

For hunger and fullness throughout the meal day, there was a significant effect of food consumption for both hunger and fullness (*p* < 0.001), but no effect of condition, group or interactions (*p* > 0.05 for all; data not shown). For SQ at lunch, there was a significant effect of condition (*p* < 0.001), with SQ at HCHO being greater than HFAT (Fig. 3), but no effect of group or condition and group interaction (*p* > 0.05 for both). Full sample results can be found in Additional file 1.

Liking and wanting fat appeal bias in the hungry and fed state did not differ between conditions, nor were there any condition and group interactions (*p* > 0.05 for all; Table 3). From pre- to post-lunch, ANOVA revealed a significant main effect of food consumption for liking and wanting (*p* < 0.001), such that the preference for high-fat foods relative to low-fat foods decreased from the hungry to the fed state, but no main effect of group, condition or interaction effects (*p* > 0.05 for all; Table 3). Full sample results can be found in Additional file 1.

**Table 3:** Liking and wanting fat appeal bias scores pre- and post-lunch, and change from pre- to post-lunch

	HFAT		HCHO	
	HiPA	LoPA	HiPA	LoPA
Pre-lunch				
Liking	3.5 ± 17.1	7.6 ± 16.1	1.6 ± 19.2	5.8 ± 14.5
Wanting	10.2 ± 42.7	21.7 ± 30.5	12.8 ± 40.3	22.2 ± 28.9
Post-lunch				
Liking	−5.3 ± 13.1	−2.8 ± 16.5	−3.7 ± 14.0	−0.6 ± 13.3
Wanting	−13.2 ± 31.2	−7.1 ± 30.5	−17.5 ± 33.0	−2.4 ± 31.6
Change*				
Liking	−8.8 ± 14.5	−10.4 ± 12.5	−5.2 ± 18.0	−6.4 ± 13.2
Wanting	−23.4 ± 39.3	−28.8 ± 28.1	−30.3 ± 34.9	−24.7 ± 29.3

*p < 0.001 (main effect of food consumption pre- vs. post-lunch)

*HiPA* high level of physical activity, *LoPA* low level of physical activity

## Discussion

This is the first study to investigate satiation and passive overconsumption in individuals with high and low physical activity levels within a multi-level appetite control framework. Our data revealed distinct differences in free-living physical activity and body composition between HiPA and LoPA despite similar BMI. However, for both HiPA and LoPA, the nutritional manipulation of increasing dietary fat (and energy density) led to a similar level of passive overconsumption, with greater energy intake in HFAT compared to HCHO, without any concurrent changes in appetite sensations or preference for high-fat foods in the hungry and fed state.

### Physical activity, body fat and appetite control

It is important to emphasise the contribution of low levels of physical activity to the accumulation of body fat. We have shown in a non-obese sample that HiPA have greater fat-free mass and lower fat mass compared to LoPA at the same BMI. This supports recent data from our group that found that sedentary behaviour was positively associated with fat mass, while there was a negative association between moderate-to-vigorous physical activity and fat mass [2]. Over time, there exists a dose–response relationship between physical activity level and body weight, such that low levels of physical activity result in greater gains in body weight (i.e. body fat) [38]. An accumulation of body fat leads to insulin resistance and is proposed to be detrimental to satiety signalling [39, 40]. In inactive overweight and obese individuals, exercise training reduces fat mass [9, 41] and also alters the release of appetite-related peptides [19, 42], improves insulin and leptin sensitivity [43–45], and enhances satiety (measured by the SQ) over several hours after a meal [9]. Thus, regular physical activity could sensitize the appetite control system by driving energy intake (via an increase in resting metabolic rate and energy expenditure) but concomitantly increase postprandial sensations of satiety [46].

This study suggests that, in non-obese individuals, higher levels of habitual physical activity do not mitigate the passive overconsumption response when exposed to a high-fat meal. Interestingly, previous studies conducted in non-obese participants have shown enhanced satiety at higher levels of habitual physical activity without large differences in group characteristics in terms of BMI, eating behaviour traits and insulin sensitivity [16, 17]. Larger disturbances in the putative determinants of appetite control, including body composition, leptin, ghrelin, insulin sensitivity, control over eating, disinhibition, and food reward may be required to affect satiation and result in overconsumption. These differences in findings emphasise the importance of distinguishing between separate appetite-related processes when examining the impact of physical activity on food intake. Based on these observations, we can speculate that habitual physical activity may differentially affect the processes of satiation and satiety. While higher levels of habitual physical activity appear to enhance post-prandial satiety responsiveness, it is possible that factors other than physical activity (e.g. meal characteristics and cognitive factors) have a stronger influence on satiation. That said, it is plausible that a greater accumulation of body fat and/or lower levels of physical activity than seen in the present study may be necessary to dysregulate satiation and impact on meal size.

### Physical activity and passive overconsumption

The passive overconsumption paradigm used in this study achieved several outcomes. Firstly, increasing the fat content (and energy density) of a food led to an increase in energy intake. Secondly, non-obese individuals with similar BMI but differing in levels of physical activity have similar satiation response to meals varying in fat. Thirdly, SQ differed across the HFAT and HCHO conditions. This demonstrates that per calorie consumed, fat produced a smaller suppression of hunger at the test meal than carbohydrate. These data corroborate previous studies on passive overconsumption via weak satiation and further illustrate the importance of reducing dietary fat (and energy density) to avoid positive energy balance and ultimately weight gain [6, 13]. Not to undermine the contribution of regular physical activity to energy balance, as it is significant as discussed above, but it exemplifies that diet and activity go hand in hand. Indeed, evidence suggests that higher levels of energy expenditure (i.e. habitual physical activity) are beneficial for the regulation of energy balance [14]. A higher energy flux is also helpful in mitigating episodes of overconsumption and fluctuations in energy intake [47, 48]. For example, Murgatroyd et al. showed that imposing sedentary behaviour and an ad libitum diet containing 60% energy from fat resulted in a daily positive energy balance of approximately 1200 kcal more than a day with imposed exercise [49]. In our sample, free-living TDEE as measured by SWA was significantly greater in HiPA than LoPA (600 kcal more per day). Even when accounting for these differences in TDEE with the PO index, the response to passive overconsumption did not differ (13 vs. 16% of TDEE, respectively). This may have been because energy intake was only measured at one meal.

Previously, Caudwell et al. found that after a 12-week exercise-training intervention (5 days per week, 500 kcal per session), overweight and obese individuals significantly lowered energy intake at a high-energy-density dinner test meal (~4 kcal/g, >50% energy from fat) but not at a low-energy-density dinner test meal (~2.4 kcal/g, <25% energy from fat) [50]. Body fat status may be an important contributor to passive overconsumption as differences in energy intake between lean and obese males have been observed at a test meal following a high-fat high-energy preload compared to a low-fat low-energy preload, where the lean group subsequently compensated for the additional energy from fat whereas the obese group did not [40]. Furthermore, studies comparing appetite control between active and inactive individuals have measured satiety using preload-test meal paradigms, which led to the proposition in a recent systematic review that physically active individuals have an increased sensitivity to the energy density of foods [15]. In light of the results of the current study, in non-obese individuals, it is possible that this effect is attributable to mechanisms mediating satiety but not satiation [15].

In terms of food reward, HiPA and LoPA did not differ in their hedonic preference for high-fat foods (liking and wanting fat appeal bias score) when hungry or after eating the HFAT and HCHO meals. However, a recent study showed differences in other markers of liking and wanting using the Leeds Food Preference Questionnaire between active and inactive males; but the 2 groups were not matched for BMI and differed much more in body composition than the current study [20]. Our data showed that HiPA had a tendency for greater restraint score than LoPA, which suggests more cognitive restriction of food intake. Regardless, both groups behaved similarly at the HFAT and HCHO test meals, highlighting the strong environmental influence of dietary fat on energy intake. Independent effects of fat and energy density in passive overconsumption have been observed. It appears that energy density is a stronger driver of passive overconsumption than fat itself because when the energy density of high-fat and high-carbohydrate meals are matched, energy intake is similar [13, 51]. In fact, Hopkins et al. have recently shown independent and positive associations between energy expenditure (via resting metabolic rate) and energy density with daily energy intake [52].

### Limitations

There are a number of limitations to take into account in the present study. Firstly, passive overconsumption was measured using a single meal and limits the extrapolation of findings beyond that meal. Any compensation in the post-ingestive period remains unknown. As previous studies reported differences in satiety between active and inactive individuals [16–19], an effect might have been observed in the hours after consuming the HFAT meal, but this was outside the scope of the present study and needs to be addressed in future studies. Secondly, while objective measurement of physical activity was taken after the participants were included in the study and confirmed distinct physical activity levels between HiPA and LoPA, classification of the groups was based on the IPAQ (self-report) and might have confounded the groups. Other potential confounders not taken into account that may have also affected the results include levels of fat mass, fat-free mass, and dietary restraint. Thirdly, the relatively small number of subjects and large inter-individual variability in responses may have resulted in the study being underpowered to detect significant differences. Furthermore, while it was attempted to match the groups by sex, the final sample included a slightly greater proportion of women in the LoPA group compared to the HiPA group (57% vs. 50%, respectively), which may account for the some of the differences in body composition observed. However, when sex was added as a covariate, the significant differences fat-free mass (*p* = 0.005) and percentage body fat (*p* = 0.015) remained, as well as the trend towards a difference in fat mass (*p* = 0.068).

## Conclusions

This study provides evidence to support the beneficial effects of high levels of habitual moderate-to-vigorous physical activity (≥4 days/week) on body composition but did not reveal differences in passive overconsumption between non-obese individuals with high and low levels of physical activity matched for BMI. This may help to clarify the differential role of physical activity level in the distinct processes of satiation and satiety. While satiety appears to be enhanced with higher levels of physical activity [15], it is likely that other factors have a stronger influence on satiation. However, it still remains unknown if the lack of observed effect on satiation in LoPA extends to individuals with a greater accumulation of body fat (obese). Nevertheless, in non-obese individuals, our data suggest that a high-fat meal overpowers any physiologic or behavioural influence of physical activity level on eating behaviour, highlighting the importance of a healthy diet in maintaining adequate appetite control and body weight in an obesogenic food environment.

## Additional file

Additional file 1: Full sample results for energy intake, satiety quotient, and liking and wanting fat appeal bias score. (DOCX 16 kb)

## Abbreviations

BES: Binge eating scale
BMI: Body mass index
CoEQ: Control of Eating Questionnaire
HCHO: High-carbohydrate
HFAT: High-fat
HiPA: High level of physical activity
HOMA-IR: Homeostasis model of risk assessment – insulin resistance
LoPA: Low level of physical activity
PA: Physical activity
PAL: Physical activity level
RER: Respiratory exchange ratio
RMR: Resting metabolic rate
SQ: Satiety quotient
SWA: SenseWear Armband
TDEE: Total daily energy expenditure
TFEQ: Three-Factor Eating Questionnaire
VO_2max_: Maximal aerobic capacity
WC: Waist circumference
YFAS: Yale Food Addiction Scale
