# Supplementary material for: Impact of physical activity level and dietary fat content on passive overconsumption of energy in non-obese adults
Source: Int J Behav Nutr Phys Act. 2017 Feb 6;14:14. doi: 10.1186/s12966-017-0473-3 (PMC5294904; doi:10.1186/s12966-017-0473-3)
Supplement: Additional file 1: — Full sample results for energy intake, satiety quotient, and liking and wanting fat appeal bias score. (DOCX 16 kb) [file 12966_2017_473_MOESM1_ESM.docx]

Additional table 1. Full sample results for energy intake, satiety quotient, and liking and wanting fat appeal bias score

|  | **HFAT** | **HCHO** |
| --- | --- | --- |
| Energy intake (kcal)* | 1408.9 ± 452.3 | 1019.5 ± 287.3 |
| Satiety quotient (mm/kcal)* | 5.6 ± 2.1 | 7.4 ± 3.6 |
| **Fat appeal bias - hungry** | | |
| Liking | 5.5 ± 16.5 | 3.6 ± 17.0 |
| Wanting | 15.8 ± 37.2 | 17.4 ± 35.1 |
| **Fat appeal bias - fed** |  |  |
| Liking | -4.1 ± 14.7 | -2.1 ± 13.6 |
| Wanting | -10.2 ± 30.6 | -10.1 ± 32.8 |
| **Fat appeal bias - change hungry-fed**** | | |
| Liking | -9.6 ± 13.4 | -5.8 ± 15.6 |
| Wanting | -26.0 ± 34.0 | -27.6 ± 32.0 |

**p* < 0.001 (main effect of condition).

***p* < 0.001 (main effect of food consumption).
